# Enhanced Antitumor Immunity Contributes to the Radio-Sensitization of Ehrlich Ascites Tumor by the Glycolytic Inhibitor 2-Deoxy-D-Glucose in Mice

**DOI:** 10.1371/journal.pone.0108131

**Published:** 2014-09-23

**Authors:** Abdullah Farooque, Niharika Singh, Jawahar Singh Adhikari, Farhat Afrin, Bilikere Srinivasa Rao Dwarakanath

**Affiliations:** 1 Division of Radiation Biosciences, Institute of Nuclear Medicine and Allied Sciences, Brig. S. K. Mazumdar Marg, Delhi, India; 2 Department of Biotechnology, Jamia Hamdard University, Delhi, India; University of Missouri-Kansas City, United States of America

## Abstract

Two-deoxy-D-glucose (2-DG), an inhibitor of glycolysis differentially enhances the radiation and chemotherapeutic drug induced cell death in cancer cells *in vitro*, while the local tumor control (tumor regression) following systemic administration of 2-DG and focal irradiation of the tumor results in both complete (cure) and partial response in a fraction of the tumor bearing mice. In the present studies, we investigated the effects of systemically administered 2-DG and focal irradiation of the tumor on the immune system in Ehrlich ascites tumor (EAT) bearing Strain “A” mice. Markers of different immune cells were analyzed by immune-flow cytometry and secretary cytokines by ELISA, besides monitoring tumor growth. Increase in the expression of innate (NK and monocytes) and adaptive CD4^+^cells, and a decrease in B cells (CD19) have been observed after the combined treatment, suggestive of activation of anti-tumor immune response. Interestingly, immature dendritic cells were found to be down regulated, while their functional markers CD86 and MHC II were up regulated in the remaining dendritic cells following the combination treatment. Similarly, decrease in the CD4^+^ naïve cells with concomitant increase in activated CD4^+^ cells corroborated the immune activation. Further, a shift from Th2 and Th17 to Th1 besides a decrease in inflammatory cytokines was also observed in the animals showing complete response (cure; tumor free survival). This shift was also complimented by respective antibody class switching followed by the combined treatment. The immune activation or alteration in the homeostasis favoring antitumor immune response may be due to depletion in T regulatory cells (CD4^+^CD25^+^FoxP3^+^). Altogether, these results suggest that early differential immune activation is responsible for the heterogenous response to the combined treatment. Taken together, these studies for the first time provided insight into the additional mechanisms underlying radio-sensitization by 2-DG *in vivo* by unraveling its potential as an immune-modulator besides direct effects on the tumor.

## Introduction

Tumors show enhanced glucose usage purportedly to generate metabolic energy (ATP) and macromolecular synthesis for sustaining rapid cell proliferation [1], besides evading apoptotic cell death and defense against oxidative stress [2]. Increased dependency on glucose, the altered metabolic hallmark of cancer has been a target for developing cancer therapeutics [3], [4]. The glucose analogue 2-deoxy-D-glucose (2-DG), an inhibitor of glycolytic ATP production has been shown to enhance radiation and chemotherapeutic drugs induced damage in a number of cancer cells under *in vitro* and *in vivo* conditions, by inhibiting repair and recovery processes as well as augmenting cell death selectively in cancer cells [5]–[11]. Several *in vitro* and *in vivo* studies have indeed confirmed that 2-DG either spares or protects the normal cells and tissues from damage caused by radiation and chemotherapeutic drugs under conditions that enhance tumor cell death and local tumor control [12]–[20].

2-DG is a structural analog of glucose that selectively accumulates in cancer cells after phosphorylation by hexokinase. Enhanced/preferential death of cancer cells by 2-DG may be due to a number of reasons, including intracellular glucose deprivation, resulting in induction of stress-related proteins [21]–[22], the generation of free radicals [23], or inhibition of energy metabolism [22]–[25]. Recent clinical trials administering oral 2-DG in combination with ionizing radiation (IR) to treat malignant gliomas indicate that the combined treatment is well tolerated, provides survival advantage and better quality of life with negligible acute toxicity and protection to surrounding normal tissues [26]–[28]. However, the combined treatment of 2-DG and focal irradiation of the Ehrlich ascites tumor (EAT) in mice leads to complete response (cure; tumor free survival) in a fraction of the mice (45–50%), while a partial response (only growth delay) has been observed in the remaining (50–55%) [29]. Therefore, we hypothesized that this differential response could be due to the differences in the effects of the combined treatment on host tumor interactions mainly in the form of immune system.

Earlier studies have shown that a combination treatment of 2-DG and etoposide [a topoisomerase II poison based anticancer drug] in EAT bearing mice, which also results in a differential response does not significantly alter the CD4/CD8 ratios, suggesting that it is not selectively toxic to a given subset of lymphocytes [30]. Further, *ex-vivo* studies with mouse splenocytes and thymocytes have also shown that 2-DG delays endogenous and radiation-induced apoptosis [15]. While these studies have established that a combination of 2-DG with radiation and chemotherapeutic drugs is not toxic to the immune cells, the effects on immune cells cross talk, which may also contribute to the radio-sensitization of tumors (and heterogenous response) have not been investigated so far. Indeed, there is an intricate relationship between glucose metabolism and immune system [31]–[32] and several effects of 2-DG on cells like UPR, N-linked glycosylation of protein's etc. have also been found to influence the functional status of immune cells in several ways [33]. Therefore, it was considered worthwhile to delineate the possible cellular targets of 2-DG in immuno-regulatory networks during radio-sensitization of Ehrlich ascites tumor in mice.

In the present studies, we investigated the potential contributions of altered host response in the form of immune-modulation induced by systemically administered 2-DG in tumor bearing mice followed by focal irradiation to the tumor that resulted in either partial (tumor growth delay) or complete response (cure; disease/tumor free survival). Results convincingly show that alterations in the immune system induced by the combined treatment (2-DG + Radiation) influence the radio sensitization of EAT by 2-DG. Activation of anti-tumor immunity in the peripheral blood both in terms of increase in the levels of innate and adaptive cells and decrease in B cells has been observed after the combined treatment. Further, decrease in the CD4^+^ naïve cells which was paralleled with the increase in CD4^+^ activated cells confirmed the immune activation. Moreover, shift from Th2 and Th17 to Th1 in the form of cytokine and switching of antibody class were associated with complete response (cure).Interestingly, this immune activation or anti-tumor immune response observed after the combined treatment appears to be mainly due to the depletion in T regulatory cells (CD4^+^CD25^+^FoxP3^+^).

## Materials and Methods

### Flow cytometry antibodies and reagents

Monoclonal antibodies to mouse CD4(APC,FITC), CD8(PE), CD25(PE), CD62L(PE), CD44(FITC), CD69(APC), CD45(Per CP Cy 5.5), CD28(PE), TCR-β(PE), TCR-γδ(FITC), CD 49 b(FITC), NK 1.1(PE), CD14(APC), CD 11c(PE), MHC II(FITC), CD86(FITC), CD19(PE), CD40L(PE), Foxp3 (FJK-16s clone, PE) were purchased from e Bioscience. Golden syrian hamster IgG (PE) was used as an isotype for anti-mouse CD28 antibody. For all experiments, intranuclear staining of Foxp3 was performed using the Foxp3 Fix/Perm Buffer kit (e Bioscience). Data was analyzed using FACS Diva software.

### Experimental animals

Male Swiss albino ‘strain A’ mice of 6 to 8 weeks old (average body weight 25±2gm) maintained on standard diet (Liptin, India) and water ad libitum were selected for experimental studies from an inbred colony in the animal house of the institute maintained at a constant temperature of 22±2°C and 50% humidity under a 12-h light/dark cycles. Mice were housed in plastic cages in specific pathogen-free conditions and groups of 5–10 animals were made in one cage. After adaptation period of a week, mice were randomized by weight into 5 groups (5–12 mice per group).The animals not appearing healthy or inactive, sluggish or losing their weight were not included in the experiments. The five groups consist of (I) Normal (mice with no tumor and no treatment) (II) None (mice with tumor but no treatment, injected PBS according to the weight, vehicle control) (III) 2-DG (tumor bearing mice treated with 2-DG (2 gm/kg b.wt) alone i.v.) (IV) R (tumor bearing mice treated with focal irradiation of 10 Gy) (V) 2-DG+R (tumor bearing mice treated with intravenous injection of 2-DG followed by focal irradiation of 10 Gy to the tumor). 3–6 animals were used in groups I to IV in each experiment, while 7–12 animals were used in group V to obtain sufficient number of CR in a single experiment. Each experiment was repeated 3–4 times.

### Authorization

This study was carried out in strict accordance with the recommendations in the Guide for the Care and Use of Laboratory Animals in cancer research of United Kingdom Coordinating Committee on Cancer Research (UKCCCR). The protocol was approved by the Committee on the Ethics of Animal Experiments of the Institute of Nuclear Medicine and Allied Sciences (INMAS), Defence Research and Development Organization (DRDO) (Institutional Ethical committee number under which this study has been approved is INM/IAEC/2011/08/001). All efforts during sacrifice of animals were made to minimize suffering. Different groups of mice were euthanized using cervical dislocation at 1 day and 21 days post treatment, at which time blood and lymph nodes were collected for further immune system related studies.

### Tumor transplantation

The inbred Swiss albino strain ‘A’ male mice used in these studies were obtained from the Institutes' central animal facility and weighed 20–25gm at the time of tumor implantation. The Ehrlich ascites tumor (EAT) cells (strain F-3) obtained from Institute for Biophysics, University of Frankfurt, Germany was maintained by serial passage of tumor cell suspension in the peritoneal cavity of strain ‘A’ mice [7]. Tumors were implanted by subcutaneous injection of 9×10^6^ cells (in 0.1–0.15 ml volume) into the right hind leg. Tumor volume was calculated using the formula: V = π/6 (d1×d2×d3), where d1, d2 and d3 are the three orthogonal diameters measured with the help of calipers. Experiments were performed when the tumors had attained a volume of 300–450 mm^3^ (5–6 days after implantation). Animals were sacrificed using cervical dislocation method, when the tumor reached a volume to avoid tumor burden related discomfort to the animal as per the UKCCCR guidelines for the welfare of animals in experimental neoplasia [34].

### Treatments

Mice bearing subcutaneous tumors were held in restrainers for intravenous administration of 2-DG (2gm/Kg b.wt) through the tail vein. The injection volumes of these chemicals prepared in PBS were 0.1 – 0.15 ml. 2-DG was administered immediately before irradiation and PBS was used as a vehicle. The tumors were focally irradiated with gamma radiation to a total dose of 10 Gy at dose rate 1.8 –2.5Gy/min in a ^60^Co gamma teletharapy unit (Bhabhatron II, Bangalore, India), with a source to sample distance of 80cm and a field size of 2cm×2cm. PBS was injected in control group according to the body weight.

For radio-sensitization studies, 2-DG (2gm/Kg b.wt) was administered immediately before focal irradiation (10Gy) of the tumor as mentioned earlier [29]. Changes in the tumor volume were taken as a parameter of response. A delay in the tumor growth alone was taken as partial response (PR), while complete tumor regression coupled with animal survival beyond 200 days (without re-growth of the tumor) was considered as complete response (CR).

In order to implicate the effects of host factors two approaches were followed. In the first, 2-DG was given intra-tumorally (2-gm/kg b.wt.) rather than intravenously to minimize the effects of host factors in the immune-competent mice and intravenously in immune-compromised athymic (nude) mice. Local tumor control was observed by monitoring the changes in tumor volume and cure (if any).

### Total Lymphocyte counts and differential leucocyte counts

Blood samples were collected from the orbital plexus of different treatment group's animals at different time intervals and analyzed for blood cell populations. Briefly 100 µl of blood was collected in heparinized 1.5 ml micro centrifuged tubes. Blood was acquired on automated hematological analyzer MS-4 (Melet Schloesing laboratories, France) for different blood cell population counts.

### Staining of cell surface markers

Peripheral blood mononuclear cells (PBMCs) were isolated to study the different innate and adaptive immune cell markers. Briefly, blood was collected in heparinized vials to prevent clotting. Further blood was loaded on histopaque in the ratio 2∶1 and centrifuged at 1800 rpm for 20 minutes at 37°C. Buffy coat present at the interface of two layers was collected and washed twice with PBS. PBMCs were then stained for several immune cells using anti mouse golden Syrian hamster IgG, NK 1.1, CD49b, CD19, CD14, CD4, CD8, CD62L, CD44, CD28, CD11c, MHC-II, CD86, CD69, CD45 and CD40L antibody purchased from e-Biosciences. After the antibody addition the samples were kept at room temperature for 1 hr. Further, the samples were washed twice with PBS and re-suspended in flow staining buffer. The samples were then acquired and analyzed on BD LSR II using FACS diva software.

### Cytokine estimation and antibody class switching analysis

At different time intervals post treatment, blood samples were collected from orbital plexus of the mouse eye and serum was isolated. Briefly, the blood was kept at room temperature for 1 hr and then centrifuged at 6000 rpm for 20 min at 4°C. Upper layer in the form of serum was taken out and stored at -20°C for further use. Cytokine estimation and antibody class switching were done according to manufacturer's protocol. Cytokine CBA kits were purchased from Bendermed systems, Austria for Th1, Th2 and Th17 cytokines and antibody class switching ELISA kit from ICL technologies.

### Intracellular transcription factors analysis by flow cytometry

PBMCs were isolated from blood as mentioned earlier. Cells were suspended in the 100 µl of flow staining buffer. Anti- mouse CD4 antibody was then added to this suspension and incubated for 1 hr at room temperature. Cells were then washed twice with PBS and fixed in 200 µl fixation buffer for overnight at 4°C. The fixed cells were washed and incubated with 500 µl permeabilization buffer (1X from e Biosciences) for 30 minutes at room temperature. The suspension was then centrifuged and the pellet was incubated with 100 µl permeabilization buffer and 2 µl rat serum for 15 minutes at room temperature. The fluorescently labeled antibodies against FoxP3 were added and then incubated for one hour. The mixture containing antibodies was washed with PBS two times, the final pellet re-suspended in 500 µl flow staining buffer and analyzed on flow cytometer.

### Statistical Analysis

Unless indicated otherwise, data are expressed as means ±SD. The significance of differences between groups was determined by a Student *t* test or one-way ANOVA followed by Newman-keuls multiple comparison tests using Graphpad prism software. For each test, the experimental unit was an individual animal.

## Results

### (1) Host factors (immune system, T cells) contribute to the complete cure elicited by the combined treatment (2-DG+R)

**Figure 1:**
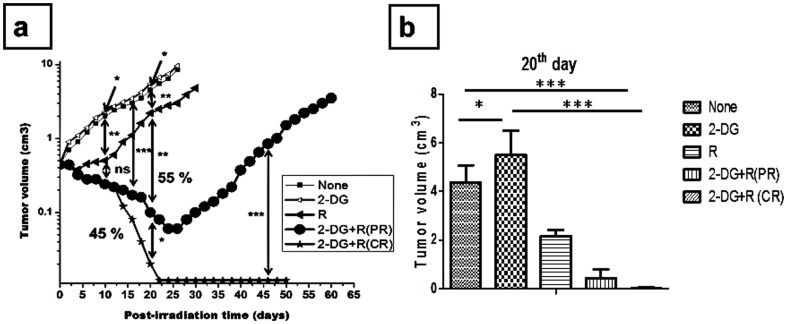
Effects of 2-DG on radiation induced changes in the volume of Ehrlich ascites tumor in mice. (a) Tumor volume was measured up to 60 days in animals receiving the combined treatment (2-DG + radiation), while in other groups animals were sacrificed according to ethically permissible tumor burden. (b) Tumor volume in different treatment groups 20 days post treatment. Values are averages (±1SD) of three independent experiments (n = 27-32). SD values (≤10% of the mean) are not visible as they are lesser than or equal to the size of the symbols. (*P<.01, **P<.001, ***P<.0001, ns- non-significant).

Focal irradiation of the tumor resulted in growth delay (P<.001), which was further enhanced by the administration of 2-DG (2-gm/kg b.wt.; i.v.) (P<.0001). The combined treatment elicited complete response (CR) by way of cure (tumor free survival) in ∼45% of the animals and partial response (PR) in 55% of animals where tumor re-growth was observed after initial regression, suggesting intergroup heterogeneity in the tumor response to the combined treatment of systemic 2-DG and focal irradiation of the tumor (Fig. 1) (P<.0001). Interestingly, the administration of 2-DG (i.v) alone marginally enhanced the rate of tumor growth (P<.01).

**Figure 2:**
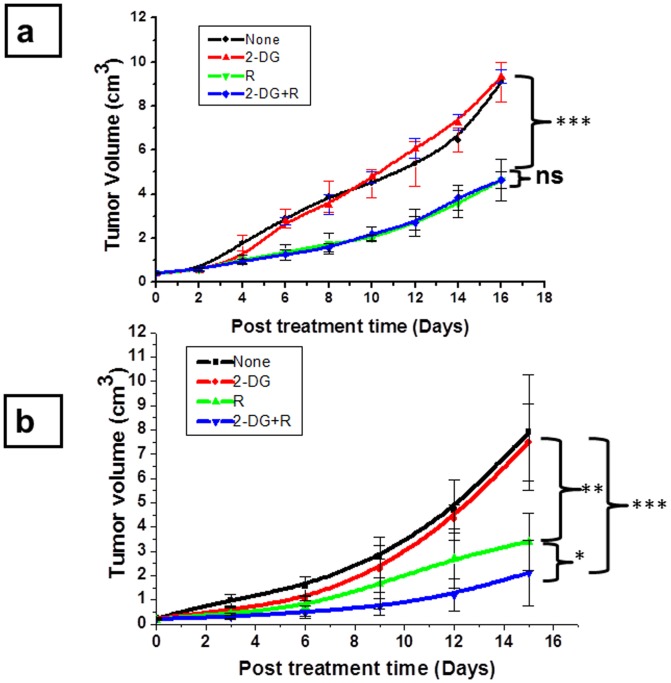
Immune system contributes to the complete response by the combined treatment (2-DG and radiation) in tumor bearing mice. (a) In this approach, 2-DG was administered intra tumorally (2gm/kg b wt.) in immune competent mice to minimize the contribution of circulating factors and focal irradiation of tumors was carried out with a total dose of 10 Gy. (b) In an alternative approach, nude mice were used and 2-DG (1gm/Kg b wt.) was administered intravenously with focal irradiation of the tumor. Results presented are average (±1SD) of three independent experiments (n =  24-30) (*P<.01, **P<.001, ***P<.0001, ns- non-significant).

Heterogeneous response (CR and PR) elicited by the combined treatment (2-DG + radiation) among identically treated mice prompted us to investigate the potential role of host factors comprising immune system in determining the local tumor control under these conditions. Towards this end, we minimized the effects of the systemic factors by adopting two approaches. In the first, 2-DG was administered intra-tumorally rather than intravenously (systemically) in the immune-competent mice and in the second we used immune-compromised (athymic; nude) mice and administered 2-DG intravenously (i.e. systemic). A lack of complete response (cure) following the combined treatment (2-DG + radiation) was evident in both these approaches (Fig. 2a&b), in contrast to 45% regression observed in the immune competent mice that received the combined treatment with intravenous 2-DG. However, in nude mice, the extent of tumor regression was higher in the combined treatment as compared to radiation alone (Fig. 2b) (P<.01), which was not observed following intra-tumoral administration of 2-DG in the immune competent mice (Fig. 2a) (P = ns).These results suggest that host factors mainly in the form of immune system have a role in tumor response to the combined treatment, particularly in determining the complete response (cure). Therefore, we systematically investigated the effects of 2-DG and radiation on the immune status by monitoring/analyzing various indicative parameters at different time points after the treatments. Observations made at 24 h (1 day) after the treatment to study the effects of treatments on the immune system (at that time tumor volumes were almost same in all the treatment groups) and at 21 days, when the complete and partial responders become apparent and immune-modulation could be due to the tumor burden.

### (2) Lympho-depletion correlates with complete cure

**Figure 3:**
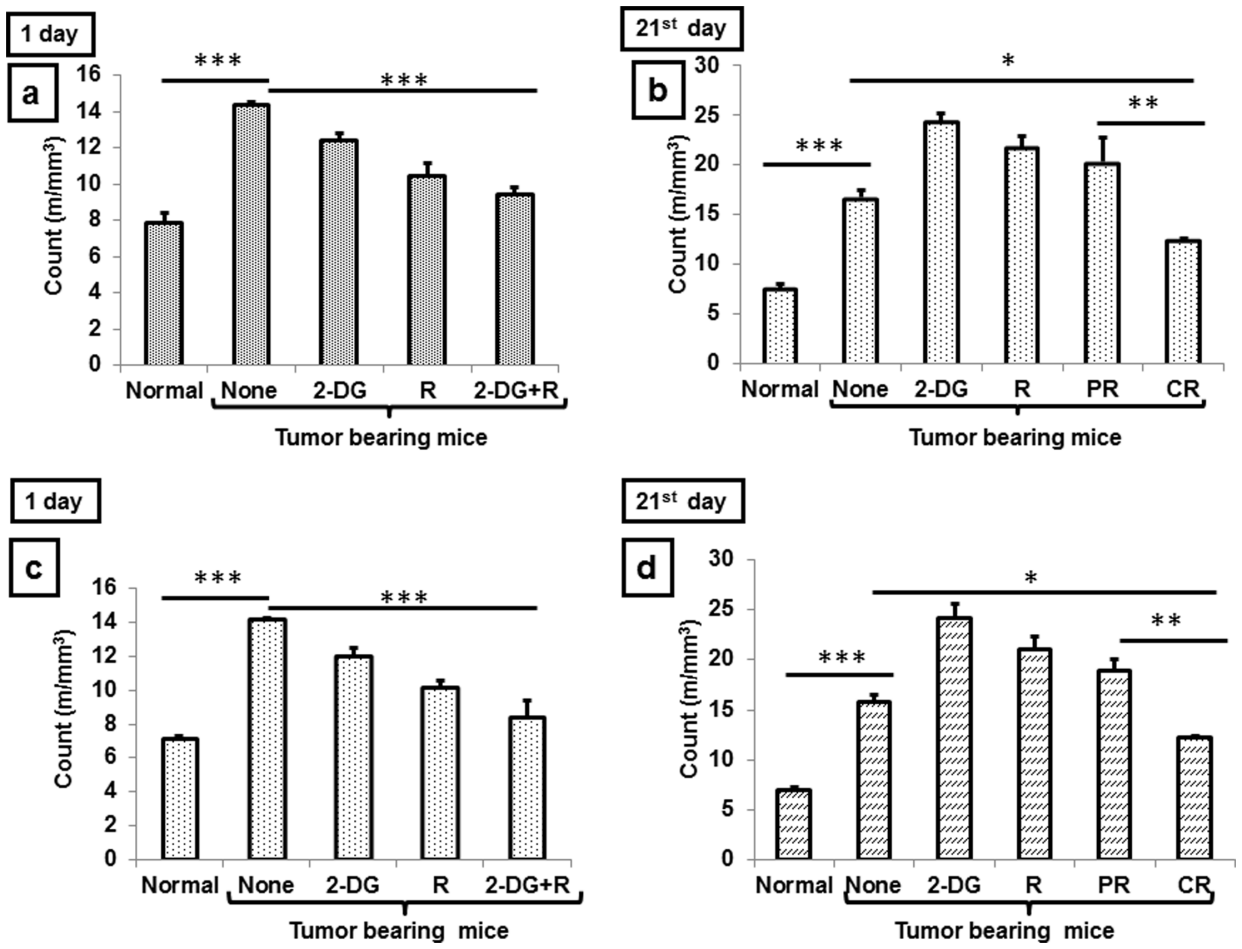
Lymphodepletion correlates with the complete response to the combined treatment of 2-DG and radiation. Blood was collected from orbital plexus in heparinized vials and analyzed for total (TLC) and differential leucocyte counts (DLC) for WBC at (a) 1 day and (b) 21 day and for lymphocytes at (c) 1 day and (d) 21 days. Results presented are average (±1SD) of four independent experiments (n = 20-24)(*P<.01, **P<.001, ***P<.0001). Normal here represents for normal mice.

It is well established that different sub-sets of lymphocytes are recruited to the tumor site as well as in the periphery due to inflammation during the cancer progression [35], [36]. Lympho-depletion results in maintaining the homeostasis favoring reduction in inflammation and in the generation of antitumor immune response. In line with these observations, we found reduction in white blood cells (WBCs) following the treatments at 1 day, with the maximum depletion observed in the combination (P<.0001, Fig. 3a). However, 21 days after the treatment mice with CR showed a higher level of WBCs depletion as compared to PR (P<.001,Fig. 3b). Similar pattern was observed in lymphocytes level as well after 1 day (P<.0001, Fig. 3c), and 21 days (P<.001, Fig. 3d). These results suggest that the extent of lympho-depletion at the end of 1 day after the combined treatment is an important determinant of the tumor response determining/influencing partial or complete (cure) response.

### (3) Up-regulation of innate and adaptive cell signaling markers during radio sensitization by 2-DG

**Figure 4:**
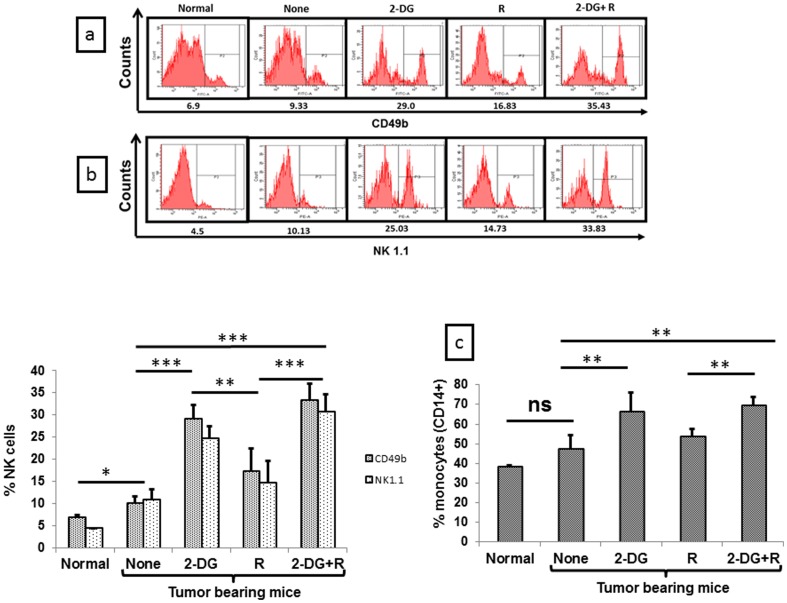
Treatment induced up-regulation of innate immunity revealed by antigenic markers in peripheral blood mononuclear cells (PBMCs); (a) CD49b and (b) NK 1.1 (NK cells); (c) CD14 (monocytes). Values shown are average (±1SD) of four independent experiments (n = 16-20) (*P<.01, **P<.001, *** P<.0001, ns- non-significant). Normal here stands for normal mice.

Since cells of the innate immunity play an important role in determining the anti-tumor immune response at the early times following perturbations of the immune system [37], [38], we investigated the effects of different treatments on innate immune cells viz. NK cells using various markers (NK1.1 and CD49b). Results clearly showed an up-regulation of NK cell markers in tumor bearing mice as compared to normal mice (P<.01, Fig. 4a,b) which was further up-regulated post treatment (CD49b and NK 1.1; Fig. 4a, b). The increase was highest in mice receiving the combined treatment (P<.0001), followed by 2-DG alone (P<.0001), while a moderate increase was seen with radiation (P<.01). A significant up-regulation of monocytes (CD14), a part of the innate immune system was also observed following 2-DG (P<.001) and the combined treatment (P<.001) while no changes were observed with radiation alone (Fig. 4c). These results suggested an activation of innate immunity immediately following the administration of 2-DG and the combined treatment, which may have contributed in initiating the initial antitumor immune response signaling and subsequent activation of adaptive immune system leading to the local tumor control and cure.

**Figure 5:**
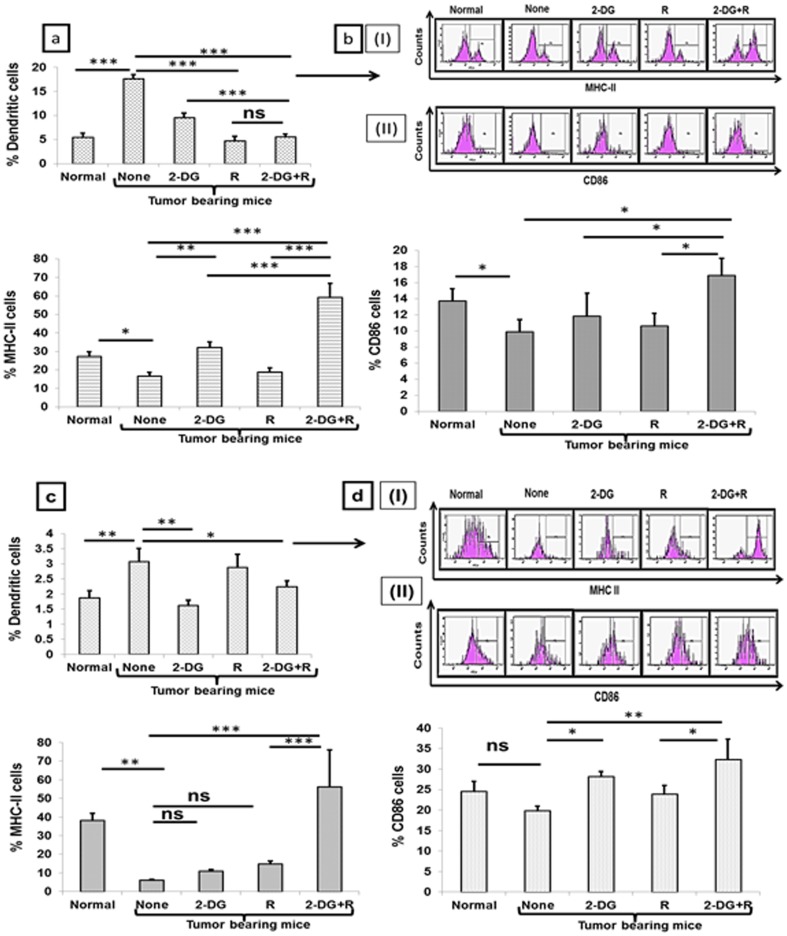
Enhanced expression of antigen presentation markers correlate with the tumor regression induced by 2-DG plus radiation. PBMCs were stained for CD11c one day after treatments, and analyzed by flow cytometry. High CD11c cells were gated and expression of MHC II and CD86 was analyzed. (a) high CD11c cells in PBMCs (b[i]), MHC II cells (b[ii]) and CD86 cells. Similarly, lymph node cells were stained for expression of MHC II and CD86 in cells with CD11c. (c) high CD11c cells in lymph node (d[i]) MHC II cells (d[ii]) CD86 cells. Results presented are average (±1SD) of three independent experiments (n = 21-27) (*P<.01, **P<.003, *** P<.0002, ns- non-significant). Normal here stands for normal mice.

Dendritic cells are known to induce specific immune response, by way of presenting antigens to autologous T cells. These cells are considered as a part of both innate and adaptive immunity or in other words these are present on the interphase of innate and adaptive immune system [39]. Maturity of dendritic cells during the tumor progression undergoes alterations resulting in the increase of immature dendritic cells leading to a reduction in the antigen presentation or tolerance to the tumor cells [40], [41]. These immature dendritic cells are one of the evasion mechanisms imparted by the tumor on the host for the tolerance induction [42]. The level of dendritic cells was clearly up-regulated in tumor bearing mice as compared to normal mice (P<.0002, Fig. 5a and 5c). Although all treatments resulted in the depletion of dendritic cells in the peripheral blood, a higher degree of depletion was observed with radiation (P<.0002) and the combined treatment (P<.0002, Fig. 5a). However, a significant increase in the levels of functional molecules MHC II (P<.003, Fig. 5b i) and CD86 (P<.01, Fig.5b ii) was observed only in combination. Similarly depletion of dendritic cells was observed in the lymph node following treatment with 2-DG and the combination (Fig. 5c). Interestingly, MHC II (P<.001, Fig. 5d i) and CD86 (P<.003, Fig. 5d ii) were maximally up-regulated following the combined treatment only. These results suggest that the combined treatment (2-DG+radiation) not only decreased the immature dendritic cells, but also enhanced the antigen presenting ability of the remaining dendritic cells in blood as well as the lymph node.

**Figure 6:**
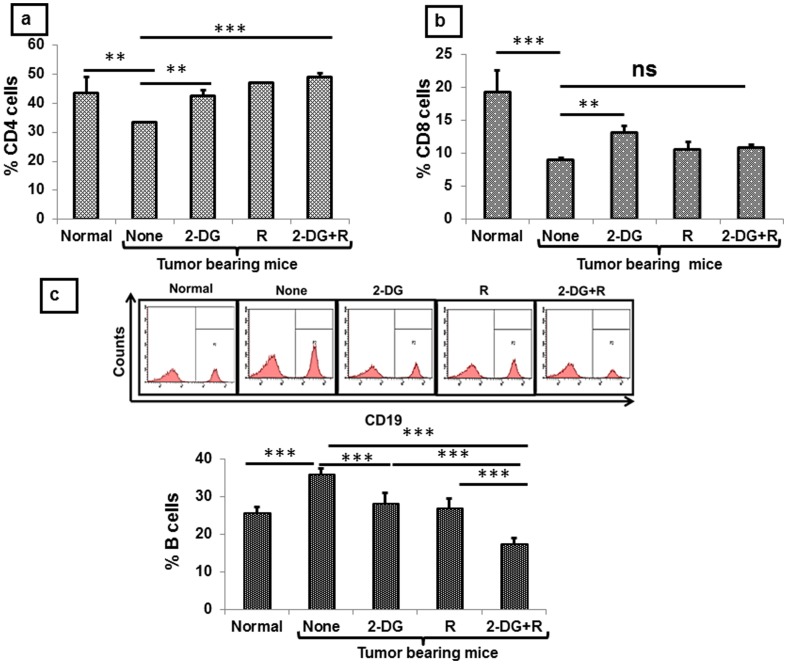
Combined treatment of 2-DG and radiation leads to the restoration of CD4 cells in the peripheral blood, besides decrease in the B cells. One day after the treatment PBMCs from blood were isolated and stained for CD4, CD8 and CD19 markers and analyzed by flow cytometry in gated lymphocytes. (a) CD4 cells (b) CD8 cells and (c) CD19 positive cells. Results presented are averages (±1SD) of three independent experiments (n = 21-27) (**P<.001, *** P<.0001, ns-non-significant). Normal here stands for normal mice.

**Figure 7:**
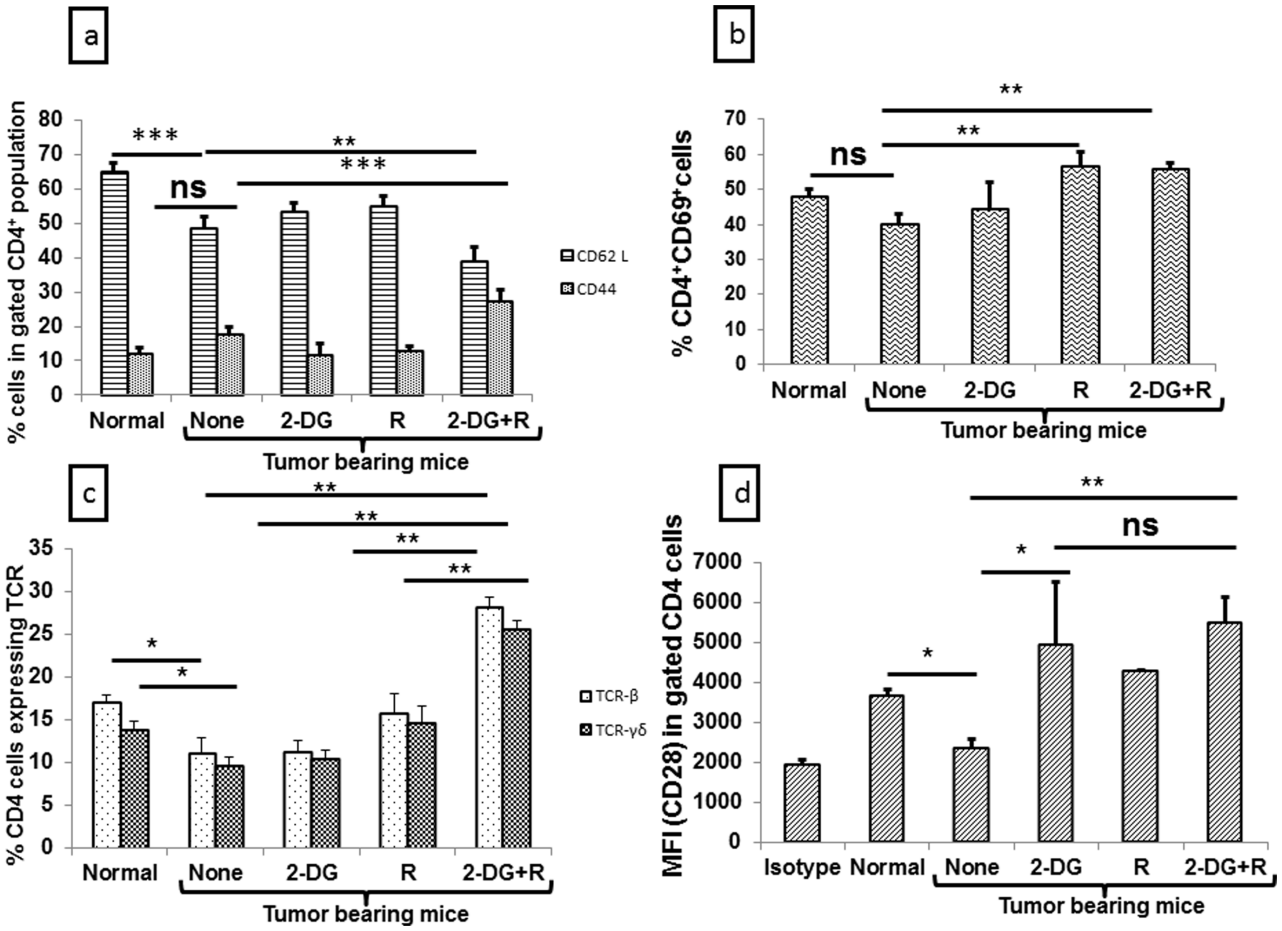
Up-regulation of signaling markers on CD4+ cells after the combined treatment (2-DG + radiation) in the peripheral blood of tumor bearing mice. PBMCs were stained for various markers (naïve and activated cells, early activation, TCR and co stimulatory molecules), one day after the treatment. (a) CD44^+^ and CD62L^+^ cells in gated CD4 cells (b) CD4^+^CD69^+^ cells (c) TCR positive cells in gated CD4 cells (d) Mean fluorescence intensity of CD28 in gated CD4 population. These results are the averages (±1SD) of three different experiments (n = 21-27) (*P<.02,**P<.003, ***P<.0006, ns- non-significant). Normal here stands for normal mice.

Adaptive immune response, the other arm of anti-tumor immune response [43], is activated by the innate immune system and after activation it helps in providing cytokines to other immune cells for proper maturation and activation [44]. Among the cells that comprise the adaptive immune system, CD4^+^ and CD8^+^ cells are vital with functional relevance as CD4 provide help to other immune cells, while CD8 can act as a cytotoxic cell to the tumor. To investigate the possible role of adaptive immunity (including cell mediated and humoral immunity) in the immune modulation brought about by 2-DG and radiation, we analyzed the status of CD4^+^ and CD8^+^ cells for cell mediated immunity and CD19 (B cell) for humoral immunity using appropriate markers. As expected, the levels of CD4^+^ and CD8^+^cells were clearly low in tumor bearing mice as compared to the normal mice (P<.001, Fig. 6a,b), while an increase was noted in the B cells (P<.003, Fig. 6c), which was maximally decreased after the combined treatment (P<.0006, Fig. 6c). No significant change was observed in the percentage of CD8 cells following the combined treatment, while a significant increase was observed in CD4 cells (P<.0006) (Fig. 6a). Further, functional activation of these CD4 cells was also observed following radiation (P<.003) and the combined treatment (P<.003) as revealed by both early activation marker (CD69) [Fig. 7b] as well as late (CD45) activation marker (data not shown). Interestingly, the combined treatment resulted in a decrease in the naive cells CD4 cells (P<.003), while increasing the level of activated cells CD4 cells (Fig. 7a, P<.0006). Further to study the TCR mediated signaling and proper activation in terms of co-stimulatory signals, we examined the status of different markers of TCR and co-stimulatory molecules and found that both radiation and the combined treatment increased the levels of TCR-β and TCR-γδ, with maximum up-regulation following the combined treatment (P<.003, Fig. 7c). Under these conditions, up-regulation of co stimulatory molecules like CD40L (data not shown) and CD28 were also observed on the CD4^+^ cells (P<.003, Fig. 7d) indicating the specific activation of CD4^+^ cells a part of cell mediated immunity in animals receiving the combined treatment while B cells a part of humoral immunity was reduced.

### (4) Th2 to Th1 shift correlates with complete cure

**Figure 8:**
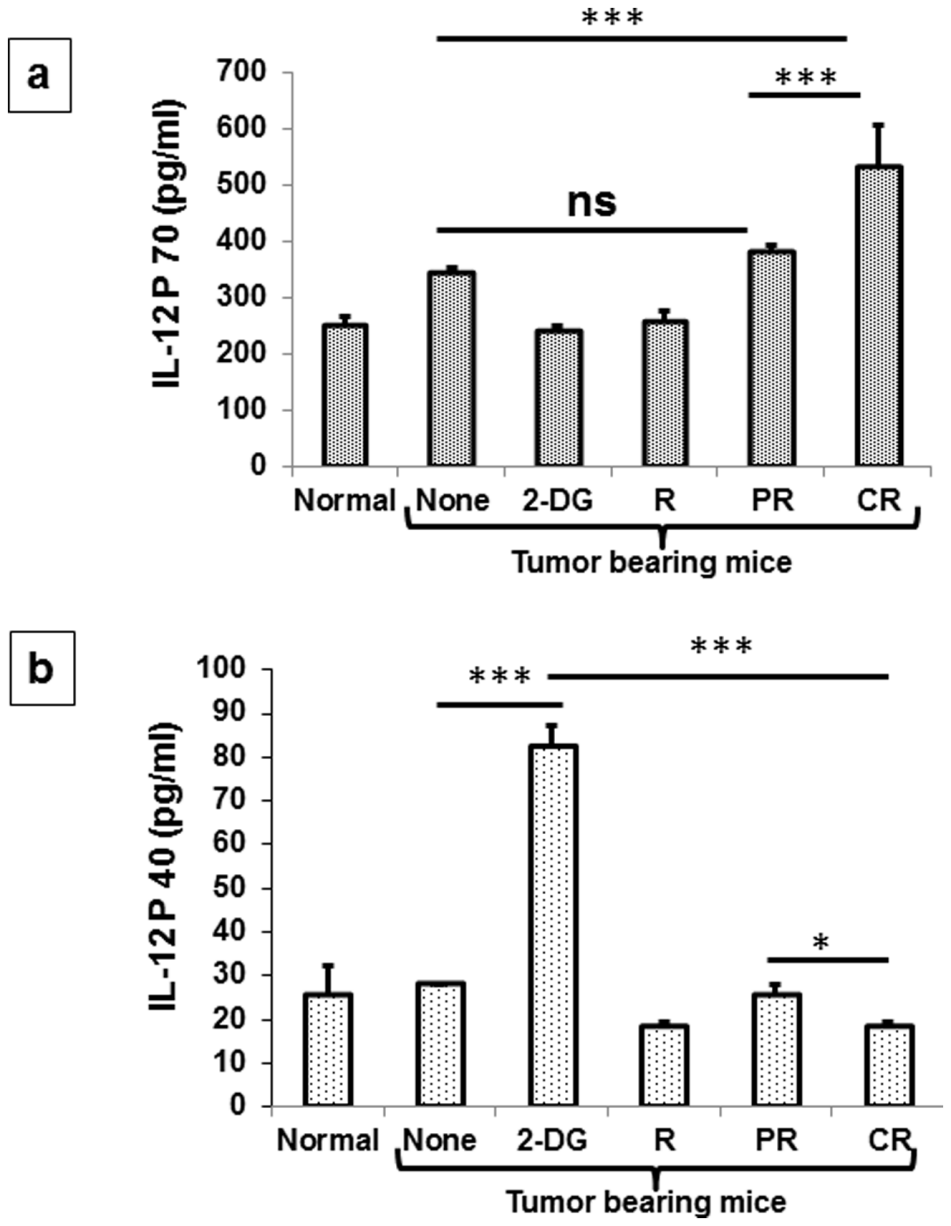
Combined treatment (2-DG + radiation) enhances the secretion of IL-12 in favor of anti-tumor immunity. 21 days after the treatment, serum was isolated from blood and cytokine levels were detected as pg/ml with the help of ELISA. (a) IL-12 P70 (b) IL-12 P40. Results presented are averages (±1SD) of three independent experiments (n = 27-36) (*P<.01, **P<.001, *** P<.0001, ns- non-significant). Normal here stands for normal mice.

**Figure 9:**
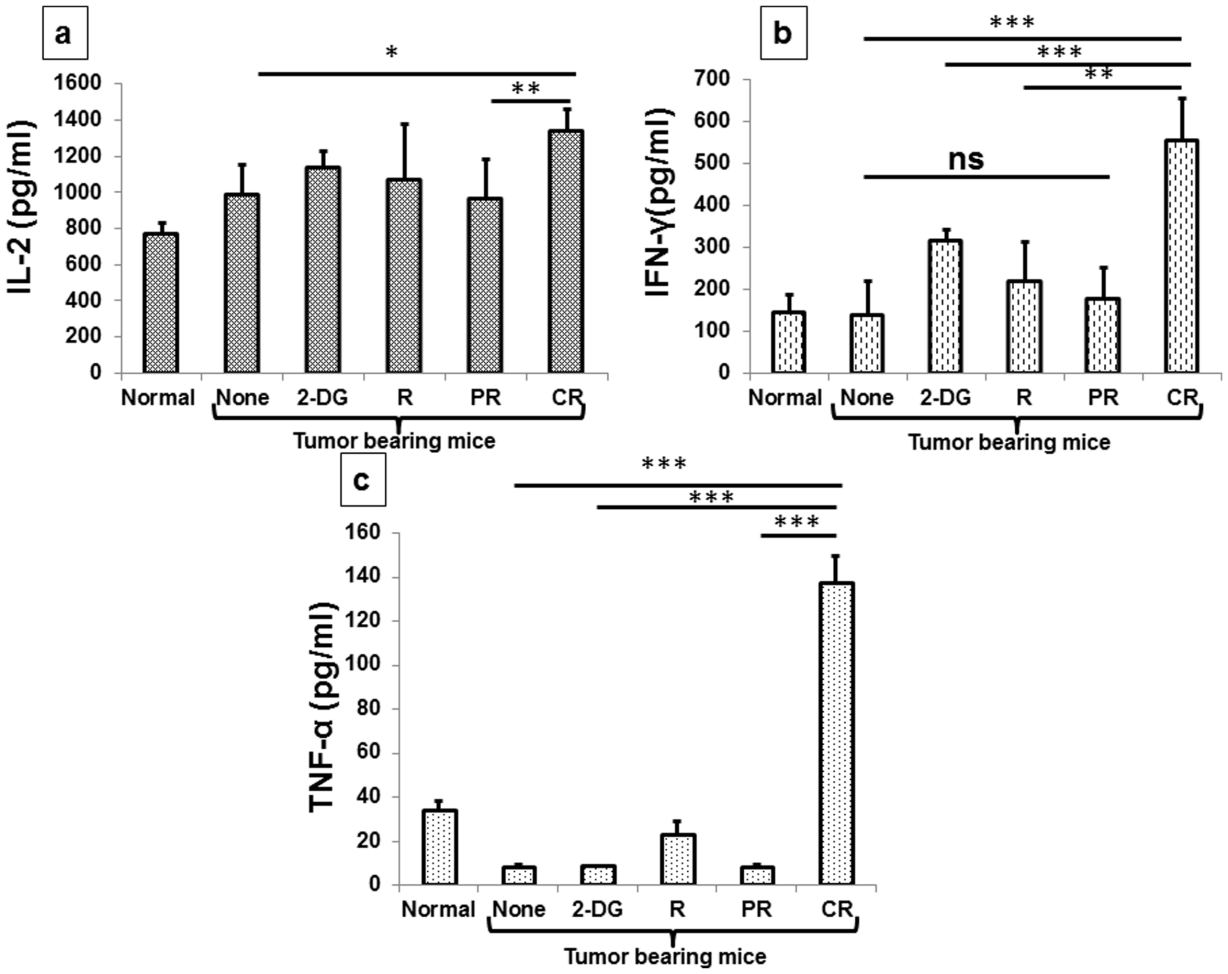
Enhancement in the Th1 related cytokines production by the combined treatment (2-DG + radiation) in tumor bearing mice. One day and 21 days after treatments, cytokine levels were analyzed in serum with the help of cytokine bead array. (a) IL-2; (b) IFN-γ; (c) TNF-α. Values presented are averages (±1SD) of three independent experiments (n = 21-27) (*P<.03, **P<.001, *** P<.0007, ns- non-significant). Normal here stands for normal mice.

IL-12 is considered as a pleiotropic cytokine and is known to be secreted by several cells including macrophages and neutrophils and helps Th0 cells to differentiate in to Th1 cells [45]–[48]. Likewise, up regulation of IL-12 P70 has been noted 1 day after the combined treatment (2-DG + radiation) (P<.02, Fig.S1 b), while the levels were differentially expressed in complete (high) and partial responders (low) at 21 days after the treatment (P<.0001, Fig. 8a). Further, the levels of IL-12P40, which is a known negative regulator of IL-12P70 were inversely correlated with the levels of IL-12 P70 as it was down regulated initially following the combined treatment(P<.009, Fig.S1 a) with differential levels in CR and PR, opposite to IL-12 P70 (P<.01, Fig. 8b). These observations strongly suggest the activation of Th1 immunity *in vivo* as a potent contributor to the radio-sensitization by 2-DG. Th1 cytokine profiling carried out under these conditions showed a significant up-regulation of IL-2 (P<.03, Fig. 9a), IFN-γ (P<.0007, Fig. 9b) and TNF-α (P<.0007, Fig. 9c) in CR as compared to PR, suggesting thereby that the Th1 biased immunity could be due to the up-regulation of IL-12.

**Figure 10:**
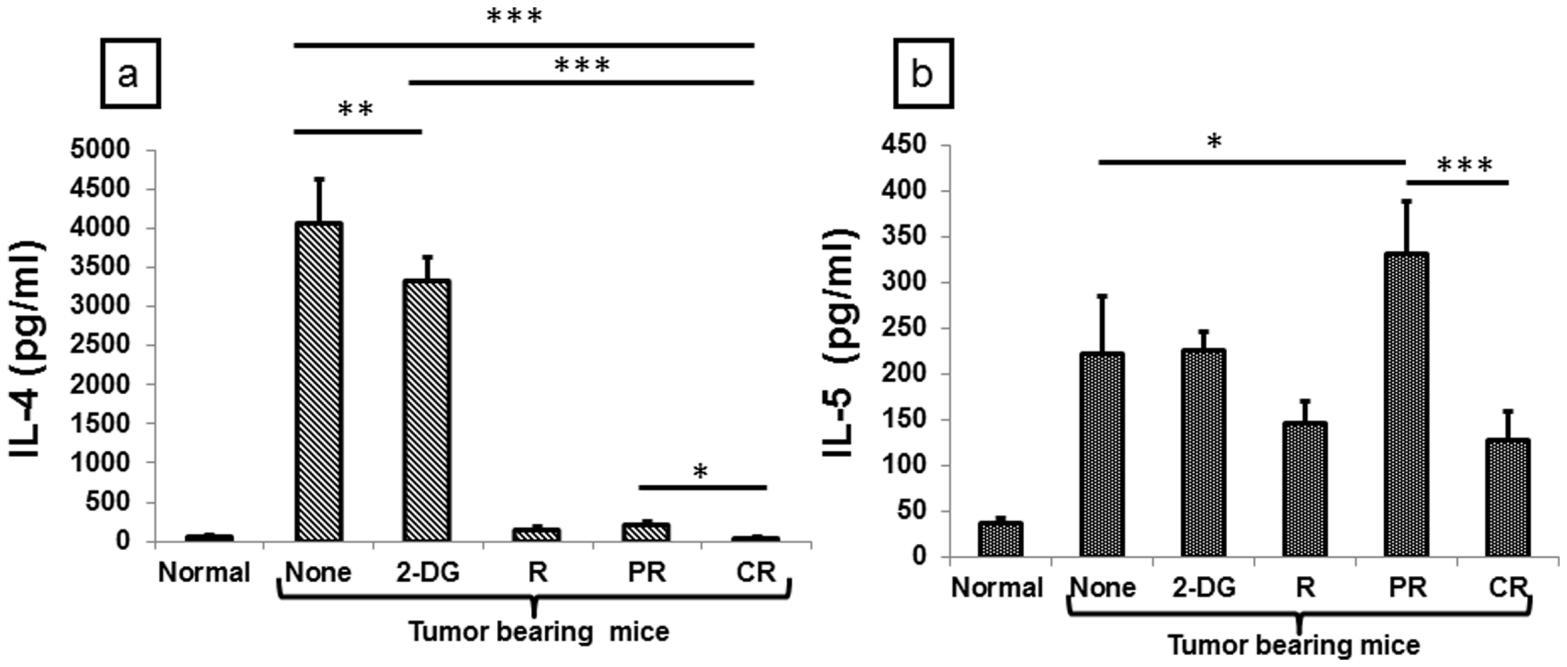
Down regulation of Th2 related cytokines in tumor bearing animals induced by the combined treatment (2-DG + radiation) observed 21 days after the treatment. Cytokine levels were analyzed in the serum with the help of CBA. (a) IL-4; (b) IL-5. Results are averages (±1SD) of three different experiments (n = 21-27) (*P<.01, **P<.001, *** P<.0001). Normal here stands for normal mice.

Th1 and Th2 cytokines cross regulate each other in order to induce a specific type of biasness [49]. To investigate the effects of various treatments on this cross talk, we carried out the Th1 and Th2 profiling in serum and found that Th2 cytokines were down regulated both at 1 day (data not shown) and 21 days after the combined treatment. However, the decrease in IL-4 (P<.01, Fig. 10a) and IL-5 (P<.0001, Fig. 10b) was significantly lower in CR as compared to PR at 21 days, suggesting down-regulation of Th2 biased immune response under these conditions. Collectively, these results suggest that the combined treatment of systemic 2-DG administration plus focal irradiation of the tumor causes a shift from anti-inflammatory response to specific anti-tumor pro-inflammatory response in mice showing cure.

**Figure 11:**
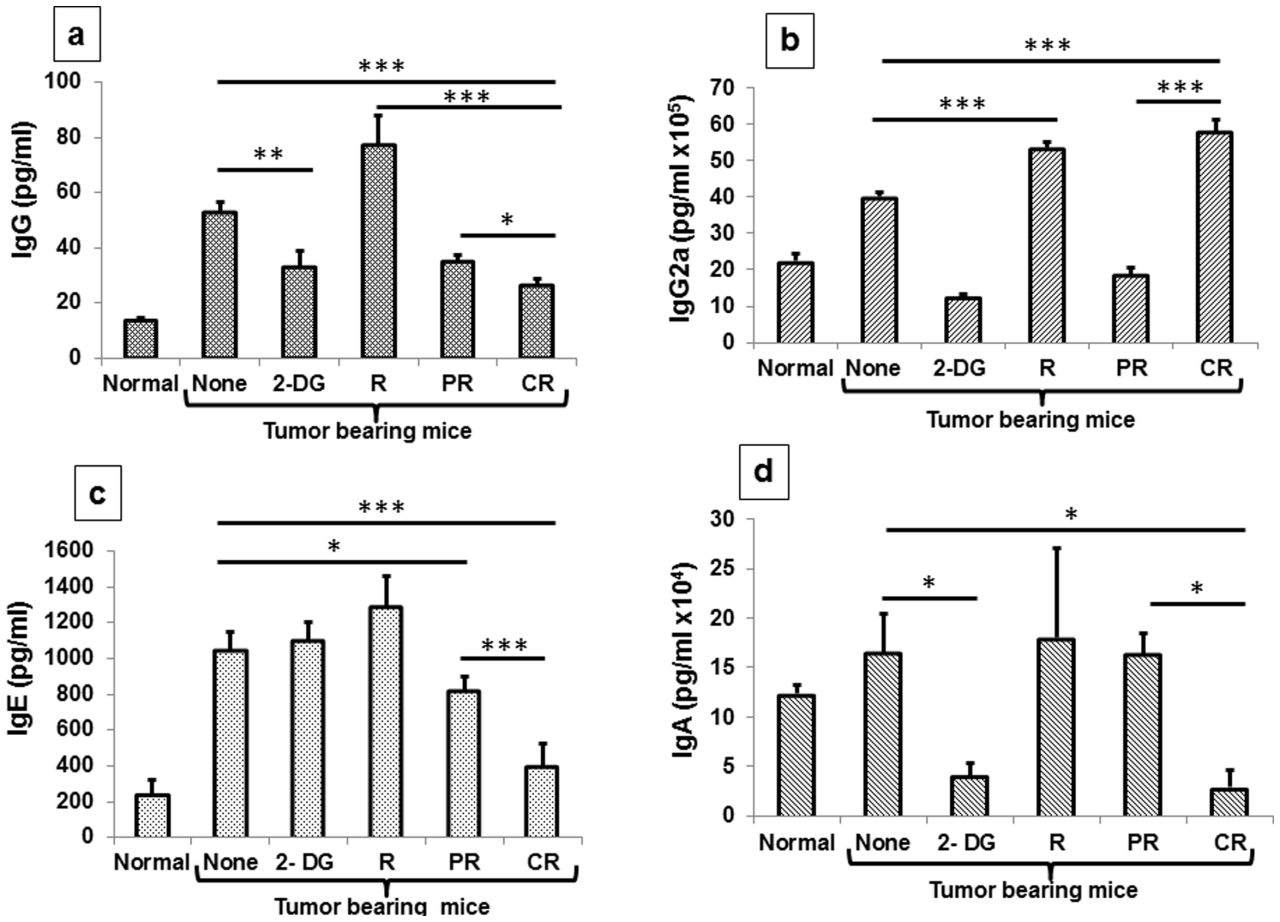
Combined treatment (2-DG + radiation) shows Th1 biased antibody class switching in complete responders. 21 days after the treatments antibody isotypes levels were analyzed in serum with the help of ELISA. (a) IgG; (b) IgG2a; (c) IgE (d) IgA. Results presented are averages (±1SD) of four independent experiments (n = 24-30) (*P<.02, **P<.001, *** P<.0001).). Normal here stands for normal mice.

Antibody class switching is a mechanism that predominantly takes place during the cancer progression and different classes of antibodies are generated because of different Th cytokines. Each class of antibody represents a specific type of Th response favoring or inhibiting tumor progression. Focal irradiation of the tumor and systemic 2-DG administration either individually or in combination significantly reduced the total IgG although maximum reduction was seen immediately (1 day) following irradiation of the tumor (P<.0001, Fig.S2 a). Interestingly, IgG2a levels were up regulated maximally in the combination after 1 day (P<.0001, Fig.S2 b). Likewise, maximum up regulation was observed in radiation as well as CR group of the combined treatment after 21 days (P<.0001), while a reduction was observed in 2-DG and PR group of the combined treatment (P<.0001) (Fig. 11 b). This up-regulation correlated well with the increase in the IFN-γ, a known inducer of IgG2a. IgE levels were down-regulated significantly in 2-DG and the combined treatment 1 day after the treatments (P<.0001, Fig.S2 c), while maximum reduction was observed in complete responders (P<.0001), correlating with the reduced Th2 response as revealed by the cytokine profile (Fig 11 c). Interestingly, significant changes in IgE were not observed following irradiation, unlike Th2 cytokines where decrease in IL-4 and IL-5 were observed (Fig. 11c). IgA antibody class known to be induced by the T regulatory cell cytokine IL-10 and TGF-β was down regulated maximally in 2-DG and the combined treatment (P<.001, Fig.S2 d), 1 day after the treatments, while 2-DG and CR groups showed maximum down-regulation (P<.007, Fig. 11d), suggesting a possible reduction in the T regulatory related cytokines not only at initial time points but also at later time points. Antibody class switching observed here are in line with the changes in cytokine levels, which also suggest a shift from Th2 to Th1 and down regulation of T regulatory cell related cytokines in mice receiving the combined treatment and showing cure.

### (5) Down-regulation of inflammatory cytokines is associated with complete cure

**Figure 12:**
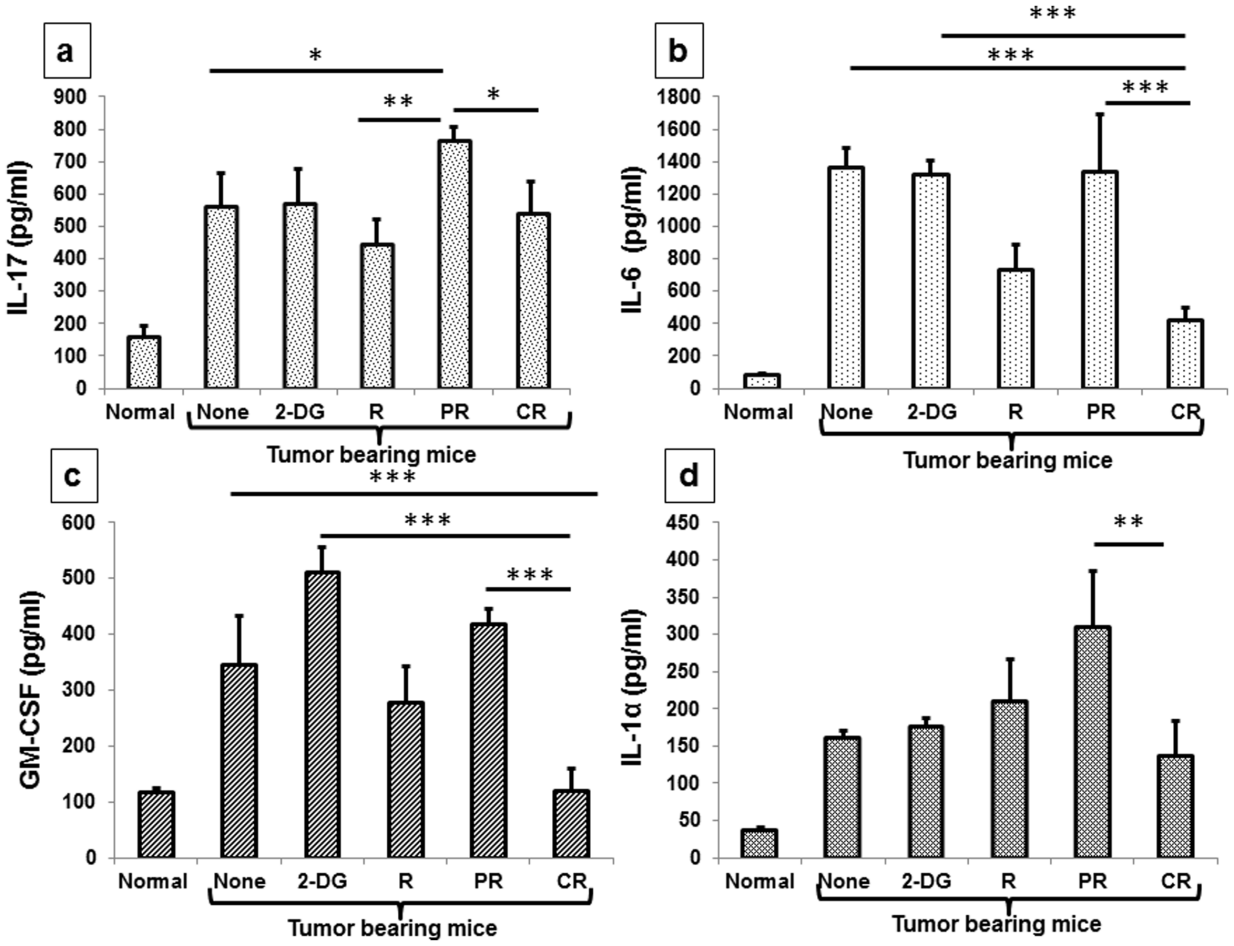
Abrogation of inflammatory response may also contribute to the cure induced by the combined treatment (2-DG + radiation) in tumor bearing mice. 21 days after the treatment, cytokine levels were analyzed in serum with the help of CBA. (a) IL-17; (b) IL-6; (c) GM-CSF; (d) IL-1α. Results presented are averages (±1SD) of three different experiments (n = 21-27) (*P<.01, **P<.001, *** P<.0001).). Normal here stands for normal mice.

Chronic inflammation is considered to be one of the major causes of carcinogenesis [50] and several cytokines play important roles in mediating the tumor induced inflammation. The outcome of their interplay leads to aberrant immune activation (also termed as inflammation) or acts as an inducer of negative feedback mechanism including generation of immune suppressive mechanisms [51]. We found a significant down regulation of inflammatory cytokines (IL-17 and IL-6) with irradiation alone and the combined treatment at 1 day (data not shown) as well as 21 days after the treatments with the levels being significantly lower in CR (P<.01, P<.0001 respectively), as compared to PR (Figure 12 a & b). Interestingly, in partial responders, the levels of inflammatory cytokines were found to be significantly higher than in the untreated group (P<.01). Similar changes observed in IL-6 suggest that the decrease in IL-17 is possibly due to a decrease in IL-6 as reported earlier [52]. Moreover, GM-CSF and IL-1α were also found to be lower in radiation and CR groups (Fig. 12 c, d) complimenting the reduced inflammation observed in the CR group. These results suggest that the combined treatment (2-DG + radiation) not only activates the immune system specifically but also reduces the inflammation.

### (6) Depletion of Tregs is correlated with the complete response

**Figure 13:**
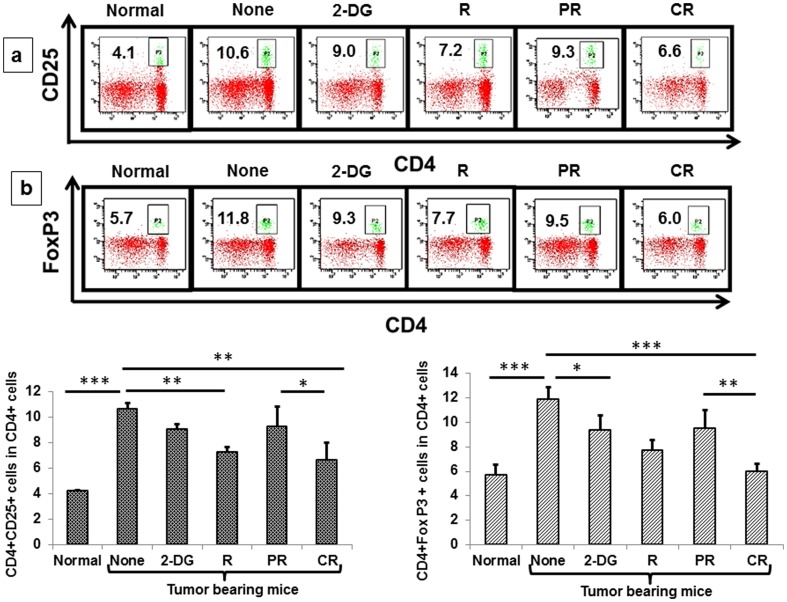
Down regulation of T regulatory cells and subsequent immune activation correlates with the complete response induced by the combined treatment (2-DG + radiation). T regs markers like CD4, CD25 and FoxP3 were studied in the PBMCs by flow cytometry (a) CD4^+^CD25^+^ and (b) CD4^+^FoxP3^+^ (%) in total CD4 cells 21 days after the treatments. Results presented are averages (±1SD) of three independent experiments (n = 36-42) (*P<.01, **P<.001, *** P<.0001). Normal here stands for normal mice.

T regulatory cells (Tregs), a subset of CD4^+^ cells profoundly influence the immune system in tumor bearing mice, by suppressing both innate and adaptive arms of the immune system [53]. Activation of Tregs is an evasion mechanism imparted by the tumors on the host to escape the immune system [54]. Tregs express several markers like CD25, GITR, FR4 etc, and the most reliable marker is FoxP3. Increase in peripheral Tregs were evident 6–7 days post implantation as compared to normal mice and were significantly up-regulated at 28 days (i.e. 21 days post-treatment) (P<.0001, Fig. 13a,b). The levels of CD4^+^CD25^+^ cells in the peripheral blood were reduced at 1 day with all the treatments, however, and maximum depletion observed following the combined treatment (data not shown). At the end of 21 days post treatment, maximum decrease was noted in radiation and CR group of the combined treatment (P<.001 (Fig. 13b). We also carried out the CD4^+^Fox P3^+^ profiling in peripheral blood and found maximum decrease in the radiation and CR group of the combined treatment (P<.001, P<.0001 respectively, Fig. 13a) at 21 days post treatment. Taken together, these observations suggest that reduction of Tregs induced by 2-DG which was accompanied by the immune activation appears to be an important contributing factor for radio-sensitizing effects by 2-DG.

## Discussion

In spite of advancements in the mechanism based targeted therapies, inter individual variations in the response to treatment remains a major limitation in achieving complete cure in many subjects. The glycolytic inhibitor 2-DG has been suggested as a potent adjuvant to radio- and chemotherapies for enhancing the efficacy as it has been found to selectively increase radiation and chemotherapeutic drug induced death of tumor cells with minimal/negligible toxicity to normal cells *in vitro*, animal tumors and in human subjects [3], [4], [5], [7], [8], [16], [18], [19], [20]. However, heterogeneity in the local tumor control observed following the combined (2-DG + radiation) treatment (Fig 1 and ref [29]) in the form of complete response (cure; tumor free survival) in 45% and partial response in 55% of the animals suggested that variations in the host factors, mainly the immune system may play an important role in the tumor response to this combined treatment. We hypothesized that early and delayed modulation of immune components by the glycolytic inhibitor might be responsible for the therapeutic response besides the direct effects on the tumor cells.

Accumulating evidences suggest that effects on the immune system significantly contribute to the efficacy of anti-cancer treatments, besides their direct effects on the tumor cells/tissues [55]. The developing tumor progressively compromises the immune system by tumor related/derived factors, which include TGF-β, IL-10, PGE2, and gangliosides [56]. This alteration depends both on the phenotype of the tumor (immunogenicity) as well as the host and facilitates the local tumor regression, growth and also metastasis [57]. Therefore, agents that have the potential to restore host immunity have been investigated as immunotherapeutic as well as adjuvant to other therapeutic modalities. Differential responses in the local tumor control induced by the combined treatment of systemically administered 2-DG and focal irradiation of the tumor (Fig 1; 45% complete response vs 55% partial response) were suggestive of differences in the effects on the immune system linked to host and tumor response. Absence of this differential response when 2-DG was either administered intra-tumorally in immune competent mice or systemically in nude mice (Fig 2) lent further support to this proposition. Since stimulation of glycolysis has been implicated in the activation of immune system [31], [32], it is reasonable to expect that this stimulation is susceptible to modifications by glycolytic inhibitors like 2-DG, which appears to differ among individual animals.

Lympho-depletion is generally considered as a beneficial phenomenon after certain types of therapies (for example adoptive cell therapy) and known to hamper the inflammation induced by cancer. Resetting of this homeostasis, which is altered during carcinogenesis, favors anti-tumor immunity [35], [36]. Increase in the innate and adaptive cells induced by the combined treatment (2-DG plus radiation) in spite of overall decrease in the lymphocytes (Fig. 3c and 3d); suggests a differential susceptibility of different cell populations to this treatment. These findings indicate that although the combined treatment leads to overall lympho-depletion, this is accompanied by specific activation of antitumor immunity, which may contribute to the cure. It is pertinent to note that the treatment regimen used here was a single fraction of 2-DG administration (i.v) followed by focal irradiation of tumor that resulted in immune alterations and tumor responses. There appears to be a strong interaction between 2-DG induced effects on the immune system (both systemic as well as tumor associated) caused immediately after the treatment (1 day) and the release of tumor (response) related factors that appears to result in progressive immune alterations leading to either complete or partial local tumor control (Fig 1).This may involve damage-associated molecular patterns in the form of immunogenic peptides generated from treatment induced acute tumor cell death (necrotic) as well as modifications of dendritic cell function [58], [59]. How this initial interplay differs among the population that results in diversified progressive responses with time is not understood and challenging to unravel.

Innate immune system is not only involved in the killing of tumor cells, but also helps the adaptive immune system for its activation [37], [38]. Interestingly, the up-regulation of innate immunity in the form of NK cells induced by 2-DG was prominent, although significant but marginal increase was also observed with radiation (Fig. 4a). However, a synergistic effect was observed with the combined treatment, suggesting that stimulation of the innate immune system in peripheral blood by radiation is accentuated by the 2-DG (Fig. 4a). Furthermore, the extent of depletion of dendritic cells in PBMCs was essentially similar with radiation and the combination, although interestingly, the functionality appeared to be higher with the combination as indicated by the levels of markers MHC II and CD86 (Fig. 5b, 5d). Taken together, these results suggest that the extent of depletion of dendritic cells may not be the major determinant of the response, but it is perhaps the functionality of the remaining dendritic cell population.

Effects of 2-DG on the immune system in tumor bearing animals have not been investigated thoroughly so far, although multiple injections has been shown to partially compromise the immune system in normal mice [60]. To the best of our knowledge, this is the first report showing that a single dose of 2-DG (2gm/kg b.wt) with or without focal irradiation of the tumor activates immune system in mice. However, 2-DG alone did not provide local tumor control despite immune stimulation (Fig 1) [29]. Recent studies have also shown that inhibition of glycolysis using 2-DG in tumor bearing animals results in the activation of CD8^+^ memory cells and antitumor immunity [61]. In line with these results, levels of CD8^+^ cells were increased at 1 day after 2-DG administration, but had no effect on the tumor growth, while significant changes were not seen with the combined treatment that resulted in cure. These discrepancies may arise due to the different tumor types, dose of 2-DG, time window and routes of administration. These findings indicate that changes in CD8^+^ levels seen 1 day after the treatment may not be a good predictor of the therapeutic response provided by the combination (2-DG + radiation) (Fig. 6b).

During the cancer progression, CD4^+^ cells undergo apoptosis leading to the impaired CD4^+^and CD8^+^cells mediated antitumor immunity [62] and therapies which restore the CD4^+^ mediated signaling are known to act as immune activators. In line with this notion, our results clearly show that the combination of 2-DG and radiation elicits anti-tumor immune response by not only restoring the levels of CD4^+^ cells, but also the levels of molecules related to its signaling i.e. CD44, CD69, CD45, TCR-β, TCR-γδ and CD28(Fig. 6a and 7a,b,c,d). Interestingly, 2-DG alone restored the levels of CD4^+^ and other innate as well as adaptive cells like in the combined treatment, but the decrease in Th2 and Th17 response was seen only in the combination and radiation suggesting the role of radiation in decreasing the Th2 and Th17 responses. Thus restoration of CD4 mediated signaling markers and decrease in the Th2 and Th17 responses, which are seen only with the combined treatment, seem to be indispensible for eliciting complete response (cure). Therefore, it appears that 2-DG alone is not potent in mounting anti-tumor immune response that could result in cure, which might be due to the aberrant immune response or excessive inflammation on account of resetting of homeostasis after the removal of 2-DG from the circulation (as the *in vivo* half-life- is 90 min.) and linked with the tumor growth.

The anti-tumor effects of radiation arise from both direct killing of the tumor cells and also the immunomodulation mainly by changing the tumor phenotype to pro-immunogenic, without significant effect on the systemic immuno-suppression [63], [64]. Further, whole body irradiation induces pro-inflammatory responses, while focal irradiation of the tumor has been found to elicit anti-inflammatory responses that contribute to the local tumor control [65], [66]. In the present studies focal irradiation of the tumor reduced the Th2 (IL-4 and IL-5) responses through B cells and inflammation (IL-6, IL-17, GM-CSF, IL-1α) (Fig. 12 a, b, c, d), which was accentuated by 2-DG besides removing the systemic immunosuppression through depletion of Tregs. This was associated with reduction in Th2 and Th17 cells and increase in Th1 cells as well as their respective antibody classes that may strengthen the shift from anti-inflammatory (Th2) to antitumor immune response (Th1) besides decrease in inflammation (Th17). These observations in the peripheral blood further support the antitumor immune activation by the combined treatment in mice showing complete response.

Upstream to the Th1 cytokines, IL-12 cytokine family plays an important role in determining the antitumor immune response or aberrant inflammation mediated through IL-12 P40. Interestingly, 1 day after the administration of 2-DG alone, a reduction in the inflammation has been found due to down regulation of IL12 P40 (Fig.S1a), but at 21 days, this down regulation was reversed and that could be linked to the excessive inflammation and aggressive tumor growth (Fig. 1, Fig. 8b). However, this reduction in P40 as observed initially (1 day; (Fig.S1a) after the combined treatment was not restored by 21 days in animals showing CR (Fig. 8b). Taken together, these results suggested that up-regulation of Th1 cytokines in the animals showing cure is due to the IL-12 P70, while regrowth is associated with the IL-12 P40 (inflammatory subunit of IL-12).

Tregs are known to suppress the antitumor immune response during cancer progression. They not only suppress innate, but also the adaptive immune responses activated during the cancer progression [51]. Although Tregs has always been a fascinating target for cancer therapy but recently management of Tregs came in to the picture rather than depletion to avoid the excessive recruitment of newly formed Tregs which are considered to be more potent in their suppressive ability [67]. Targeting Tregs without interfering the cause of up regulation of Tregs might even lead to more inflammation and aggressive cancer progression as evident in the case of 2-DG alone (Fig. 1a).

Up-regulation of Tregs during heightened inflammation as in the case of cancer is a defense mechanism to protect the collateral damage but suppression induced by the Tregs becomes nonspecific and unknowingly also suppresses the antitumor immune response. Approaches that target the source of inflammation (source of induction of Tregs) besides decreasing (depleting) Tregs may be more efficient in enhancing the therapeutic gain, as observed here following the combined treatment (systemically administered 2-DG and focal irradiation of the tumor), where a decrease in inflammation (as revealed by the cytokines; Fig. 12) and Tregs (Fig. 13a, b) was associated with complete regression of the tumor (Fig. 1). Interestingly, targeting the tumor or inflammation alone (by radiation) did not appear to be effective as it did not influence the systemic immune suppression imparted by the suppressive cells including Tregs. Therefore, it appears that the extent of decrease in Tregs, besides the reduced inflammation is one of the major determinants of the host-tumor interactions in the local tumor control.

Taken together, results of the present studies clearly demonstrate that activation of innate and adaptive immunity induced by 2-DG through CD4 mediated signaling is further amplified by radiation in the combined treatment. Similarly, it appears that radiation mainly decreases the inflammation and Th2 response, which is further down regulated by 2-DG. The combined effects of 2-DG and radiation resulted in a shift from Th2 and Th17 to Th1 (anti-tumor immunity), which was prominent in CR and could be linked with the depletion of Tregs. Further, the differential responses (i.e CR vs PR) appears to be determined by the changes in the immune status seen at 1 day after the combined treatment, reflected in the form of depletion of total lymphocytes as well as Tregs and increase in the adaptive immune cells (mainly CD4 and its signaling markers; Fig 1, Fig. 3a and 3c, 7a and 7d, 13a and 13b). A closer examination of the relationship between these changes in the immune status and local tumor control suggests that they can also serve as surrogate markers of tumor response to the combined treatment.

Glycolysis is associated with activation of normal lymphocytes (lymphocyte activation dogma)[68].Immune activation observed in tumor bearing mice following systemic administration of the glycolytic inhibitor 2-DG appears to be out of tune with the dogma, except the lympho-depletion seen at 1 day after the treatment. Many well-known effects of 2-DG other than glycolytic inhibition like UPR response, N linked glycosylation; HIF-1α etc. may actually facilitate the immune activation in the peripheral blood of tumor bearing mice by the combined treatment (2-DG + radiation). Similar studies with the tumor infiltrating immune cells are desirable as they influence the therapeutic response more than the peripheral lymphocytes. Further studies are also warranted to provide a deeper insight into the alterations induced by 2-DG in signaling pathways involved in immune modulation, so as to gainfully deploy this glucose analog as an adjuvant to other therapies (including immune therapy), besides optimizing protocols for enhancing the efficacy of radiotherapy.

## Supporting Information

Figure S1 **Combination enhanced the secretion of IL-12.** One day after treatment, serum was isolated from blood and cytokine levels were detected as pg/ml with the help of ELISA. (a) IL-12 P40 (b) IL-12P70. Results presented are averages (±1SD) of three independent experiments (n = 27-30) (*P<.01, **P<.001, *** P<.0001). Normal here stands for normal mice.
(TIF)

Figure S2 **Combination treated tumor bearing mice shows Th1 biased antibody class switching.** One day after treatment, serum was isolated from blood and antibody classes levels were detected as pg/ml with the help of ELISA. (a) IgG (b) IgG2a (c) IgE (d) IgA. Results presented are the average (±1SD) of four independent experiments (n = 24-28) (*P<.02, **P<.001,*** P<.0001, ns- non-significant). Normal here stands for normal mice.
(TIF)
