# Supplementary figures and images for: Enhanced Antitumor Immunity Contributes to the Radio-Sensitization of Ehrlich Ascites Tumor by the Glycolytic Inhibitor 2-Deoxy-D-Glucose in Mice
Source: PLoS One. 2014 Sep 23;9(9):e108131. doi: 10.1371/journal.pone.0108131 (PMC4172770; doi:10.1371/journal.pone.0108131)

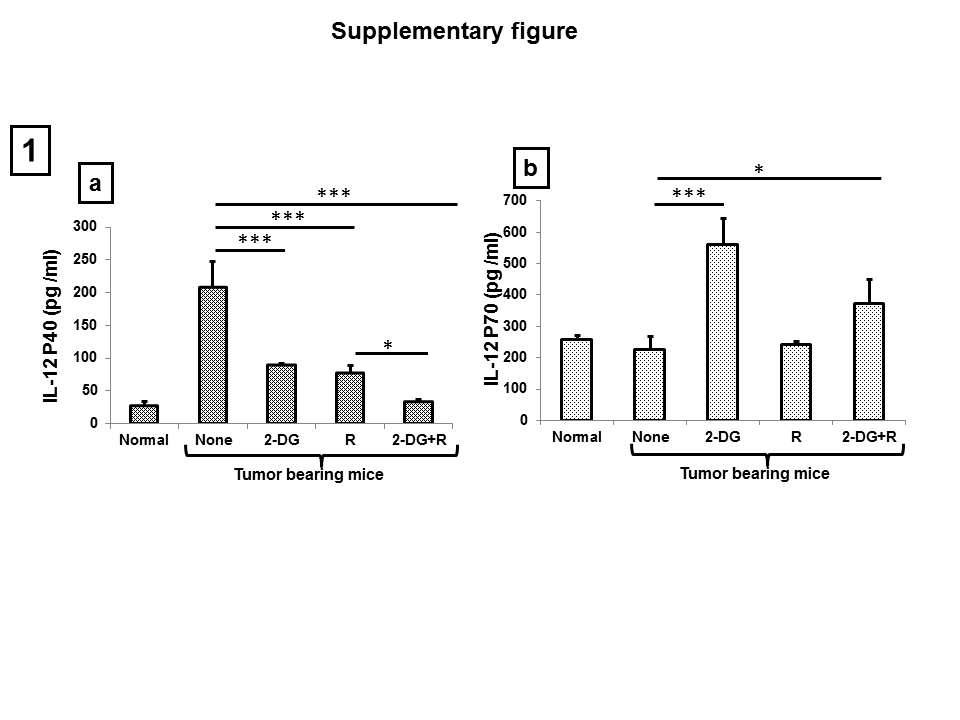

Supplement: Figure S1 — Combination enhanced the secretion of IL-12. One day after treatment, serum was isolated from blood and cytokine levels were detected as pg/ml with the help of ELISA. (a) IL-12 P40 (b) IL-12P70. Results presented are averages (±1SD) of three independent experiments (n = 27-30) (*P<.01, **P<.001, *** P<.0001). Normal here stands for normal mice. (TIF) [file pone.0108131.s001.tif]

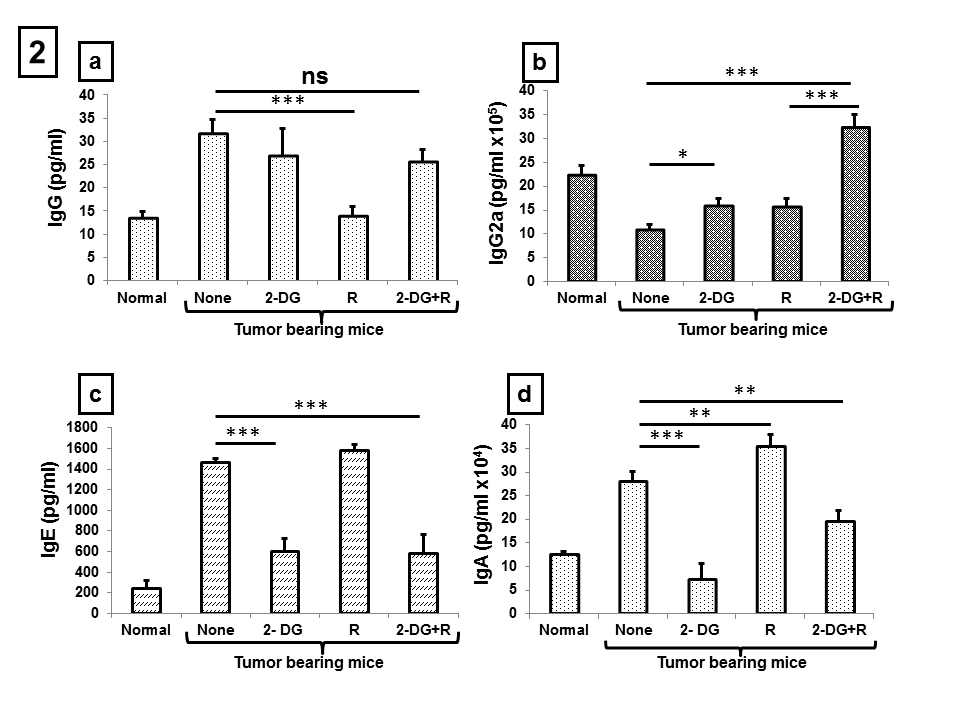

Supplement: Figure S2 — Combination treated tumor bearing mice shows Th1 biased antibody class switching. One day after treatment, serum was isolated from blood and antibody classes levels were detected as pg/ml with the help of ELISA. (a) IgG (b) IgG2a (c) IgE (d) IgA. Results presented are the average (±1SD) of four independent experiments (n = 24-28) (*P<.02, **P<.001,*** P<.0001, ns- non-significant). Normal here stands for normal mice. (TIF) [file pone.0108131.s002.tif]
